# High intra-task and low inter-task correlations of motor skills in humans creates an individualized behavioural pattern

**DOI:** 10.1038/s41598-022-24479-w

**Published:** 2022-11-23

**Authors:** Shoko Kasuga, Ethan Heming, Catherine Lowrey, Stephen H. Scott

**Affiliations:** 1 Centre for Neuroscience Studies, Queen’s University, Room 219, Botterell Hall, Kingston, ON K7L 3N6 Canada; 2 Department of Biomedical and Molecular Sciences, Queen’s University, Kingston, ON K7L 3N6 Canada; 3 Department of Medicine, Queen’s University, Kingston, ON K7L 3N6 Canada; 4 Kinarm, BKIN Technologies Ltd., Kingston, ON Canada

**Keywords:** Neuroscience, Neurology

## Abstract

Our motor system allows us to generate an enormous breadth of voluntary actions, but it remains unclear whether and how much motor skill translates across tasks. For example, if an individual is good at gross motor control, are they also good at fine motor control? Previous research about the generalization across motor skills has been equivocal. Here, we compare human performance across five different motor skills. High correlation between task measures would suggest a certain level of underlying sensorimotor ability that dictates performance across all task types. Low correlation would suggest specificity in abilities across tasks. Performance on a reaching task, an object-hitting task, a bimanual coordination task, a rapid motion task and a target tracking task, was examined twice in a cohort of 25 healthy individuals. Across the cohort, we found relatively high correlations for different spatial and temporal parameters within a given task (16–53% of possible parameter pairs were significantly correlated, with significant r values ranging from 0.53 to 0.97) but relatively low correlations across different tasks (2.7–4.4% of possible parameter pairs were significantly correlated, with significant r values ranging from 0.53–0.71). We performed a cluster analysis across all individuals using 76 performance measures across all tasks for the two repeat testing sessions and demonstrated that repeat tests were commonly grouped together (16 of 25 pairs were grouped next to each other). These results highlight that individuals have different abilities across motor tasks, and that these patterns are consistent across time points.

## Introduction

An impressive feature of our motor system is the ability to generate an enormous breadth of voluntary motor actions, from painting a picture to playing the piano. Most daily activities require the motor system to plan and execute a series of motor actions or steps to attain a behavioural goal, including the continuous use of sensory information to provide online corrections and initiate each sub-component of an action. This ability to move and interact in the world involves a highly distributed network including cortical, subcortical, brainstem and spinal processing^1–3^, as well as a musculoskeletal system that exploits a high level of redundancy with many degrees of freedom.

It is somewhat surprising then, given the breadth of possible movements and ways to generate movement, that there is often a predictable way in which people move. We have all been able to identify a friend down the street just by the way they walk or move. Previous work has highlighted the existence of movement “signatures” or “styles” which suggest that motor patterns, such as muscle activation and body motion, will vary between individuals, but that the way in which they vary are unique to each individual^4–6^. These studies have quantified our ability to identify ourselves and others simply by viewing our/their movements^7–9^. The results have highlighted that participants consistently perform certain movements with a highly similar pattern across testing time points, in some cases even years apart^10^. This suggests that within a particular task, individuals move with a certain amount of uniqueness and predictability, but the question remains as to whether and how this unique pattern translates across different motor tasks and skills.

Previous work surrounding the idea of generalization across motor skills has been equivocal. Some studies have found strong correlations between different motor tasks, which provides evidence of a generalized level of motor ability that underlies all motor skill^11–13^. However, several other studies have found weak correlations between motor skills for different tasks, suggesting high specificity in motor skills^14–17^. Historically, much of this research has been on the performance of children which includes the potentially confounding influence of motor development on the relationship between motor performances across tasks. More recently, a paper considered the performance of young adults on three tasks related to fine motor control and found no correlations between their performance on each task^17^, suggesting that even within a specific skill, there may be task-related specificity in motor ability. While some features of our motor system are constant, such as the biomechanical properties of the body, the lack of correlations across motor skills suggests the presence of highly variable neural factors related to how we plan and control our body.

The objective of this work was to (1) examine whether performance measures are correlated across several different motor tasks and (2) if the pattern of correlation is similar across time points. We tested three previously-designed upper limb motor tasks that examine (1) goal-directed motor actions, (2) rapid bimanual motor selection and action, and (3) bimanual coordination. We augmented this set with tasks that examine two other classes of motor skills. One class is repetitive actions, such as playing the drums or piano which require timing and coordination between sequential actions. Another class is fine motor skills such as writing, drawing an object or threading a needle which require precise temporal and spatial coordination. Thus, we developed two new tasks to assess: (1) speed and coordination of rapid reciprocal movements and (2) accuracy of tracking moving targets. We hypothesized that spatial and temporal measures of performance would display significant correlations for repeated tests of each task, whereas correlations across different tasks would be significantly lower. Furthermore, we used cluster analysis across all datasets (repeated tests for all individuals) to demonstrate grouping of repeated tests for each individual, providing evidence of a similar pattern of performance between test times for most individuals.

## Methods

### Participant recruitment

A total of 25 neurologically healthy individuals (16 females and 9 males, aged 19–29 years, all right hand dominant) participated in one of 2 experiments. Participants were financially compensated for their time. The experimental procedures were approved by the Queen’s University Research Ethics Board, which adhered to the principles of the Canadian Tri-council Policy statement on Ethical Conduct for Research Involving Humans. All participants provided informed consent to participate in the study and were free to stop participating in the study at any point.

### Apparatus

**Figure 1:**
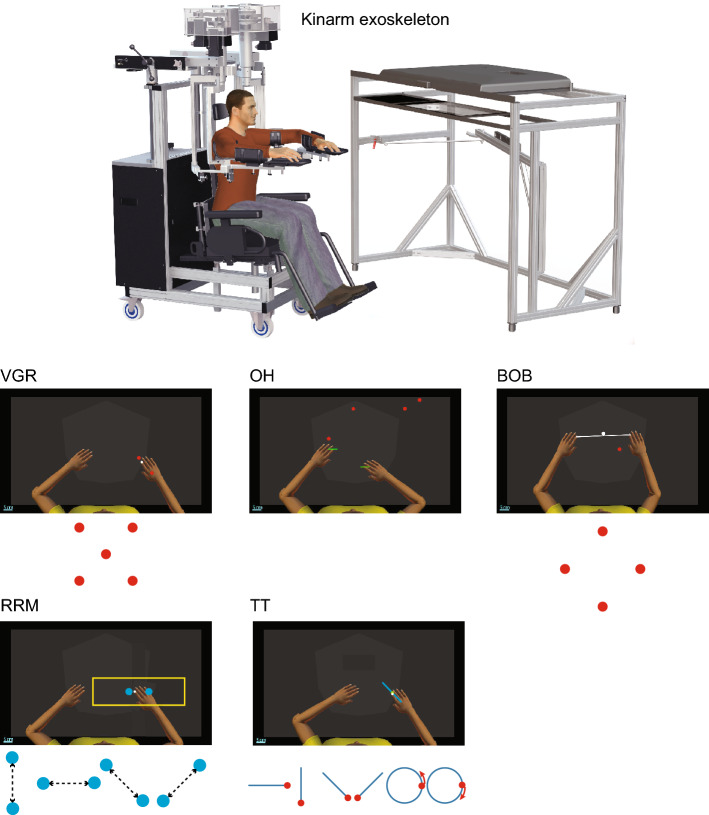
Task overview. Top panel: Schematic representation of the experimental equipment, Kinarm Exoskeleton Lab. Middle panel: Visually Guided Reaching (VGR). Participants were instructed to make reaching movements from a central target to 1 of 4 peripheral targets distributed uniformly on the circumference of a circle, presented once each in a pseudo-random order. Object Hit (OH). Virtual paddles appeared at the participant’s hand and the individual was instructed to use these paddles to hit balls that appear randomly from various locations across the top of the screen and move towards the participant. Ball On Bar (BOB). A virtual bar connecting the participant’s hands is presented on the display and a virtual ball is placed on the centre of the bar. There were 4 target circles presented to the participant in a clockwise order starting with the one closest to the participant, and the individual was asked to move the virtual ball on the bar into the target. Bottom panel: Rapid Repeated Motion (RRM). The position of the participant’s index fingertip was visible as a white cursor and the individual was instructed to move back and forth between the two red targets as quickly as possible for 15 s. There were four pairs of targets: parallel to the sagittal and frontal planes as well as in the diagonal directions. Target Tracking (TT). The position of the participant’s index fingertip was visible as a white cursor and the individual was instructed to keep the cursor in the moving target (green when the cursor is in, red when the cursor is out). Tracking paths included motion along straight lines parallel to the sagittal and frontal planes and the diagonal directions, and a circle (clockwise and counter-clockwise).

The experiment was performed using the Kinarm Exoskeleton Lab (Kinarm, Kingston ON, Canada^18,19^; Fig. 1). Participants sat in a height-adjustable chair and their arms were attached to an adjustable mechanical linkage that permitted movement of the arms in the horizontal plane. The subject was then moved into a workstation with a horizontal virtual display aligned with the workspace of the arms. A barrier under the display prevented the participants from directly seeing their arms. Torque motors attached to the linkage could apply mechanical loads to the elbow and/or shoulder joint and encoders were used to quantify motion of the shoulder, elbow and hand. Experimental set-up and robotic testing were completed by the same examiner for all participants.

Participants completed 2 repetitions of 5 motor tasks: Visually Guided Reaching (VGR), Object Hit (OH) and Ball on Bar (BOB), Rapid Repeated Motion (RRM), Target Tracking (TT). One repeat of each task was first completed in a random order, and then each task was performed a second time in a random order. Repeat tests were completed to compare the similarity of performance patterns across time points. The order of hand tested in each block was also randomized for a tasks performed separately for the left and right hand (i.e. VGR, RRM, TT).

### Motor tasks

#### Visually guided reaching (VGR)

VGR is used in many research labs to quantify eye-hand coordination and goal-directed motor performance^20–23^. Briefly, participants were instructed to make reaching movements quickly and accurately from a central target (20 mm diameter) to 1 of 4 peripheral targets (20 mm diameter) distributed uniformly on the circumference of a circle (10 cm from the center target; Fig. 1). The central target location was positioned such that it coincided with a shoulder angle of 30° and the elbow angle of 90° (full elbow extension was 0°). Participants began each trial by placing a white cursor (10 mm diameter) representing hand position within the central target for 1250–1750 ms. A peripheral target was illuminated and participants were instructed to move quickly and accurately to the target. After 1250–1750 ms, the centre light was re-illuminated and participants generated a movement back to the central target. The 4 peripheral targets were presented once each in a pseudo-random order and each target was repeated 5 times (i.e. 20 trials in total). There were 4 catch trials in which there was no target presented.

#### Object hit (OH)

Object Hit assesses the ability to perform rapid motor actions throughout the workspace^23–26^. Good performance requires the ability to generate a goal-directed motor action to a moving target, bimanual planning to select which arm to use to hit each object, and spatial awareness across the workspace. In this task, virtual 5 cm paddles appear at the participant’s hand (Fig. 1). The participant is instructed to use these paddles to hit balls that appear randomly from various locations (10 bins spanning 80 cm) across the top of the screen and move towards the participant. As the task proceeds, the number and speed of the balls increases making the task more difficult as time progresses. Haptic feedback is provided (small force pulse from the robot) when contact with a ball is made by a participant. A total of 300 balls are presented during the task.

#### Ball on bar (BOB)

The purpose of this task is to assess the ability of participants to perform bimanual motor actions^27,28^. In this task, a virtual bar connects the participant’s hands and a virtual ball is placed on the centre of the bar. The bar length was 30 cm but is modelled as a virtual spring so it can vary in length. There were 4 target circles (20 mm diameter) located 10 cm from the centre of the workspace (centre of workspace was at a shoulder angle of 30° and the elbow angle of 90°) and the targets are presented to the participant in a clockwise order starting with the one closet to the participant (Fig. 1). Participants were asked to move the virtual ball on the bar into the target as quickly and accurately as possible. Once the participant moved the ball into the target for 1 s, the next target appeared. The subject moved to as many targets as possible in 1 min.

#### Rapid repeated motion (RRM)

This task was developed to quantify one’s ability to generate rapid alternating movements. The key feature of the present task is to assess the speed and consistent timing of the transition between movements. During the task, the position of the participants’ index fingertip was visible as a white cursor (10 mm diameter circle) on the display (Fig. 1). To begin, a gray target (15 mm diameter circle) was presented in the workspace in front of the subject which turned red when the individual moved the white cursor into this target. A second red target appeared 10 cm from the first target. The locations of these targets were positioned such that the midway point (average position of the two targets) coincided with a shoulder angle of 30º and the elbow angle of 90º. After 3 s, both targets turned blue (go cue) and participants had to move back and forth between the two targets as quickly as possible for 15 s. A 15 cm × 50 cm yellow rectangle was displayed around the targets to encourage participants to stay in this area. Four pairs of targets were presented, parallel to the sagittal and frontal planes as well as in the diagonal directions, to quantify performance when coordinating different muscle groups together. The next pair of targets were presented 2 s after the completion of the previous set of targets. The order of the target orientation was fixed across participants.

#### Target tracking (TT)

The purpose of this task is to quantify one’s ability to control the position and speed of the hand to track a moving spatial target^29,30^. Tracking paths included motion along straight lines (7.5 cm) parallel to the sagittal and frontal planes, and along the diagonal directions, and finally, around a circle (10 cm diameter; Fig. 1; center of lines and circles coincided with shoulder at 30° and elbow at 90°). During the task, a stationary target appeared on the line or circle (open circle denotes start position) and the participant placed the white cursor representing the position of the index finger into this circle to initiate the task. This target turned green and after 500 ms began moving along the line or circle at 60 mm/s. For the lines, the target moved in one direction and once it returned to the end of the line, it started moving back in the opposite direction. The target moved through four complete cycles between the two targets. For circles, the target moved in one direction and completed four complete rotations, and then moved in the opposite direction for four rotations again. The objective of the task was to keep the white cursor in the moving target. The target remained green when the cursor was in the moving target and turned red when the cursor was outside of the target. The order of tracking paths displayed was fixed across participants, while the order for testing the left and right arms was randomized.

### Data processing & analysis

Position and velocity of the robot handles were recorded at a sampling rate of 1000 Hz (200 Hz for OH). Signals were filtered using a sixth-order double-pass Butterworth low-pass filter with a cutoff frequency of 20 Hz. All data were collected using Dexterit-E (version 3.7.3 or 3.8.0, Kinarm, Canada). VGR, OH and BOB form part of Kinarm Standard Tests™ and task parameters were generated by Dexterit-E (https://kinarm.com/download/kst-summary-analysis-version-3-7/).

Task data of RRM and TT were analyzed using custom scripts in MATLAB (version 2018a, Mathworks Inc., Natick, Massachusetts). Spatial and temporal parameters investigated for each task are summarized in Table 1 and some of them are visualized in the Supplementary Material (RRM, TT).

**Table 1:** Task parameter names and definitions.

Motor task	Parameter name	Parameter definition
Rapid repeated motion	Variability of movement reversal	Standard Deviation (SD) of movement reversal (i.e. the points where a subject made turns of movements). Movement reversal defined as hand position at the furthest point from the midline
	Movement distance	Distance travelled by the hand in a cycle. Mean value of all cycles is reported
	Normalized jerk index (NJ)^54,55^	Square root of the squared jerk of x- and y- dimensions normalized by movement duration and movement distance described by the following equation. Mean value of all cycles is reported. (t = timepoint) $NJ= \sqrt{1/2\times {\int }_{{t}_{start}}^{{t}_{end}}{jerk}^{2}dt\times {duration}^{5}/{distance}^{2}}$
	Cycle time	Duration of a cycle. Mean value of all cycles is reported
	Cycle time SD	Standard Deviation (SD) of duration of a cycle
	Max speed	Peak speed in a cycle. Mean value of all cycles is reported
	Dwell time^56,57^	Duration around movement reversal between the first time the speed decreases below 5% of the difference between speed minima and maxima of the preceding movement, and the first time it increases above 5% of the that for the following movement. Mean value of all cycles is reported
	Harmonicity index (H)^56,58^	Index computed by the hand acceleration (*a*) reflecting the symmetry of hand motion around movement reversal, i.e. when the hand passed midline between the target (before movement reversal) and the hand returned to the midline (after movement reversal). If there is a single acceleration peak in this time window, i.e. an ideal rhythmic movement with sinusoidal speed profile, H = 1. If there are multiple acceleration peaks, H is calculated by the following equation. Mean value of all cycles is reported $H=\mathrm{max}(\frac{{a}_{min}}{{a}_{max}},0)$
Target Tracking	Path length ratio	Ratio of the distance travelled by the hand in a trial and the distance travelled by the moving target. Mean value of all trials is reported
	Normalized jerk index	Square root of the squared jerk of x- and y- dimensions normalized by movement duration and movement distance described by the following equation. Mean value of all cycles is reported. (t = timepoint) $NJ= \sqrt{1/2\times {\int }_{{t}_{start}}^{{t}_{end}}{jerk}^{2}dt\times {duration}^{5}/{length}^{2}}$
	Target error	Mean distance between hand and target position. Mean value of all trials is reported
	SD of speed error	SD of difference between hand and target speed
	Speed maxima count	Number of hand speed maxima in a trial. Mean value of all trials is reported
	Min–max speed difference	Mean difference between pairs of adjacent local hand speed minima and maxima, for all such pairs in a trial. Mean value of all trials is reported
Visually Guided Reaching^1^	Initial direction error	Parameter definitions of Kinarm Standard Tests™ can be found online in KST Summary at https://kinarm.com/support/user-guides-documentation/
	Initial distance ratio	
	Speed maxima count	
	Min–max speed difference	
	Path length ratio	
	Movement time	
	Posture speed	
	Reaction time	
	Initial speed ratio	
	Max speed	
Object Hit^1^	Hits with D/ND	
	Hand bias hits	
	Hand transition	
	Hand speed bias	
	Movement area bias	
	Hand speed D/ND	
	Movement area D/ND	
	Hand selection overlap	
	Total hits	
	Median error	
	Miss bias	
Ball on bar^1^	Targets completed	
	Movement time	
	Ball speed	
	Hand speed D/ND	
	Hand speed diff	
	Mean bar angle	
	SD bar angle	
	Speed maxima count D/ND	
	Hand speed peaks bias	
	Bar length variability	
	Absolute reaction time difference	
	Hand path length bias	

*D* dominant arm, *ND* non-dominant arm.

For RRM, initial analysis of the data identified considerable variability in performance during the first 5 s of the first target pair. Therefore, data analysis for the very first trial (i.e. a pair of vertical targets for the first tested hand) was based on the last 10 s of the trial. We verified that subsequent trials (i.e. other pairs of targets) for the first tested hand and/or the initial pair of targets of the second tested hand were not variable across participants; therefore, the full 15 s of the trials were analyzed. A full cycle of RRM was defined as the time when the hand passed the midway point between targets until the hand passed the midway point in the same direction (i.e. reach out-and-back-and-out). For TT, the initial 1000 ms after target motion was initiated (both circle and line objects) and 200 ms after reversals (line objects only) was excluded from the analyses to remove the initial phase to initiate and catch up to the moving target.

### Correlation analysis

Each parameter was compared between Test 1 and Test 2 to compare the pattern of performance across repeat tests. To quantify test–retest reproducibility, we used a two-way, random effects intraclass correlation coefficient (ICC) for consistency. Correlations between parameters, both intra- and inter-task, were tested by Spearman’s rank correlation while correcting for multiple comparisons by controlling for the false discovery rate (FDR)^31^. This method corrects the p value at the significant level (i.e. p = 0.05) for the number of tests being performed (i.e. 2850 pairs). In order to compare the strength of each correlation, we performed a non-parametric Kruskal–Wallis ANOVA with a Bonferroni-adjusted post-hoc test.

### Cluster analysis

Hierarchical cluster analysis was used to group data for test 1 or 2 of each participant based on the standardized Euclidean distance and single-linkage method^32^. Distance between data was quantified based on a dendrogram; that is, we counted the number of nodes between the pair of data in the dendrogram. The distances were represented in a histogram and compared to the number of nodes between a randomly-generated set of data. Classical multidimensional scaling using standard Euclidean distance was used to visualize the location of each pair of tests in three dimensions.

## Results

### Test–retest reproducibility

**Figure 2:**
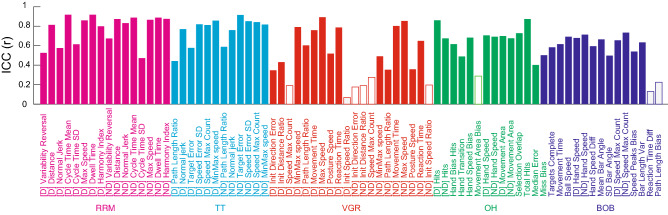
Test–retest repeatability. ICC values for all task parameters; [D] indicates dominant hand and [ND] indicates non-dominant hand. Filled bars represent significant ICC values and open bars are non-significant. Plots generated in Matlab version R2020a, https://matlab.mathworks.com.

The reproducibility of VGR task parameters was relatively low compared to the other tasks, nonetheless 4 of 10 parameters had ICC (C, 1) values above 0.70 for the dominant hand (Fig. 2). Two of 10 parameters had ICC (C, 1) values on or above 0.70 for the non-dominant hand. The reproducibility of Object Hit (OH) task parameters was high in 4 of 14 parameters (i.e. ICC (C, 1) values above 0.70). The reproducibility of Ball on Bar (BOB) task parameters was high in 2 of 14 parameters (i.e. ICC (C, 1) values above 0.70). For RRM, 5 of 8 parameters had high ICC (C, 1) values above 0.70 [good to excellent^33^] for the dominant hand. Six of 8 parameters had ICC (C, 1) values above 0.70 for the non-dominant hand. For TT, 4 of 6 parameters had high ICC (C, 1) values above 0.70 for the dominant hand. Five of 6 parameters had ICC (C, 1) values on or above 0.70 for the non-dominant hand.

### Between-parameter correlations: intra- and inter-task

**Figure 3:**
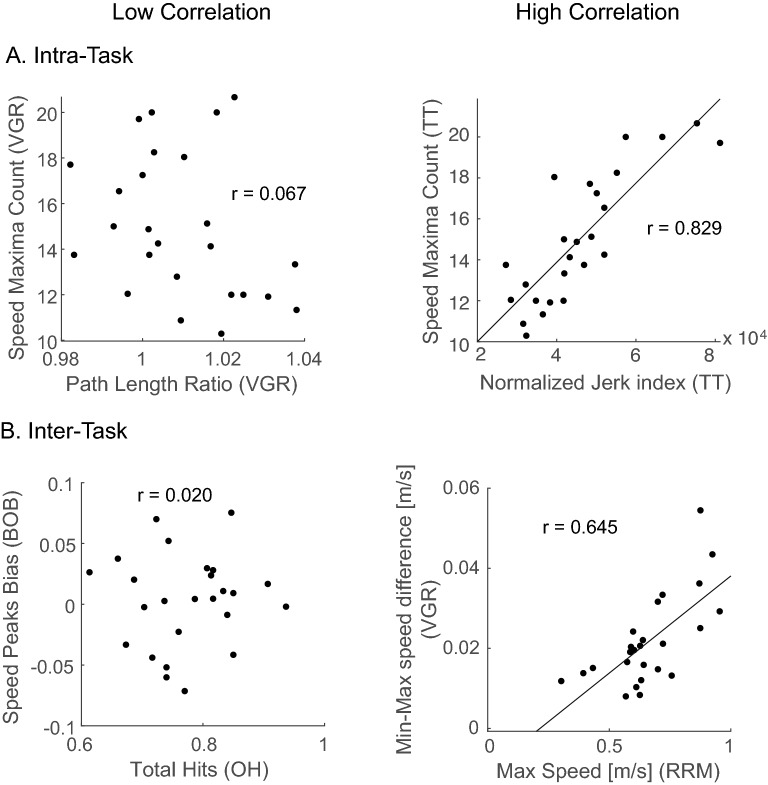
Examples of between-parameters correlations. Spearman’s ranked correlations are shown in each panel. (**A**) scatter plots of within-task parameters. Left panel shows an example of low correlation parameters (path length ratio and speed maxima count in VGR), and right panel shows an example of high correlation parameters (normalized jerk index and speed maxima count in TT). (**B**) scatter plots of parameters across tasks. Left panel shows an example of low correlation parameters (total hits in OH and speed peaks bias in BOB), and right panel shows an example of high correlation parameters (max speed in RRM and min–max speed difference in VGR). Plots generated in Matlab version R2020a, https://matlab.mathworks.com.

**Figure 4:**
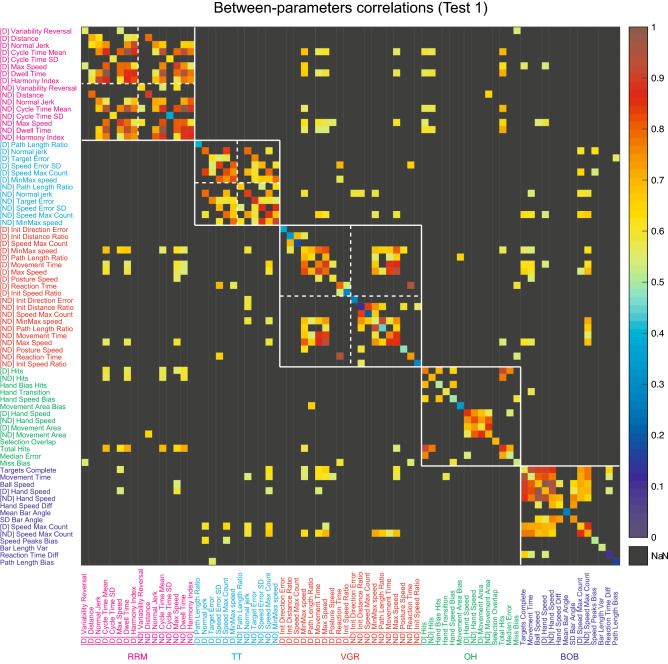
Heatmap of between-parameters correlations for all tasks. Correlations that were not significant after FDR correction are shown in black. Areas in solid white squares indicate between-parameters correlations within the same task (intra-task) and areas in dashed white squares (RRM, TT and VGR only) indicate those within the same hand trials of the same task. Diagonal axis of the heatmap shows Spearman’s correlations between test 1 and test 2 for each parameter (not corrected for significance); [D] indicates dominant hand and [ND] indicates non-dominant hand. Plots generated in Matlab version R2020a, https://matlab.mathworks.com.

Figure 3 shows scatter plots of example parameter pairings highlighting typical examples of high or low intra- and inter-task parameter correlations. Figure 4 displays a heat-map for all the intra- and inter-task parameter correlations for test 1. Areas in solid white squares in Fig. 4 indicate intra-task parameter correlations and areas in dashed white squares (VGR, RRM and TT only) indicate those within the same hand trials of the same task. Diagonal axis of Fig. 4 indicates test–retest repeatability, which will be described later. Only parameters that reached the significant level after the FDR correction are shown with coloured cells.

There is a clear difference in the pattern of intra- and inter-task correlations. In general, intra-task correlations and correlations within the same task performed with each arm, were commonly significant. Between 16–53% of all possible intra-task correlations were significant, depending on the task. Of those correlations that were significant, the Spearman’s r-values ranged from 0.53 to 0.997, with a mean r-value of 0.71. In contrast, there were far fewer inter-task correlations, with between 3–4% of all possible correlations reaching significance. Of those correlations that were significant, the Spearman’s r-values ranged from 0.53 to 0.71 with a mean r-value of 0.59.

**Figure 5:**
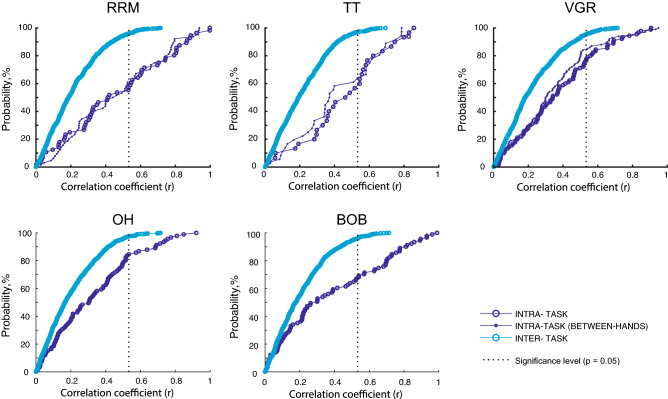
Cumulative sum plot of correlation values for each task in test 1. Blue lines with open circles indicate probability of intra-task correlations, blue lines with filled circles indicate probability of intra-task, between-hand (i.e. dominant vs. non-dominant hand test, for RRM, TT and VGR only) correlations, and cyan lines indicate probability of inter-task correlations. Dashed lines indicate significant level after FDR corrections (p = 0.05). Plots generated in Matlab version R2020a, https://matlab.mathworks.com.

Figure 5 illustrates the cumulative distribution of correlation values for each task in test 1. The plots show that correlations tend to be higher for intra-task as compared to inter-task. Interestingly, correlations between the dominant and non-dominant hands within the same task were higher magnitude than inter-task correlations for VGR, RRM, and TT. A Kruskal–Wallis ANOVA and a post-hoc test found significant difference between intra-task (same hand) and inter-task correlations (*p* < 0.001, Bonferroni-corrected), and intra-task (different hands) and inter-task correlations (*p* < 0.001); however, there was no statistical difference between the distributions within and across hands for the same task (*p* = 1.000).

The diagonal axis of Fig. 4 indicates Spearman’s correlations between test 1 and test 2. Consistent with the finding of the ICC analysis, each parameter tended to show high correlations between repeat tests. However, we found the between-parameter correlations varied somewhat for test 1 versus 2. In VGR, correlations were higher in test 2 than test 1 for the dominant hand (Wilcoxon Signed Rank test; *p* < 0.001), whereas they were higher in test 1 for the non-dominant hand (Wilcoxon Signed Rank test; *p* < 0.001). In OH, correlations were higher in test 1 than test 2 (Wilcoxon Signed Rank test; *p* = 0.045). In BOB, Correlations were higher in test 2 than test 1 (Wilcoxon Signed Rank test; *p* < 0.001). In RRM, for the non-dominant hand, median correlations were higher in test 2 than test 1 (Wilcoxon Signed Rank test; *p* = 0.003). In TT, correlations were not significantly different between the tests in either hand (Wilcoxon Signed Rank test; *p* = 0.229).

**Figure 6:**
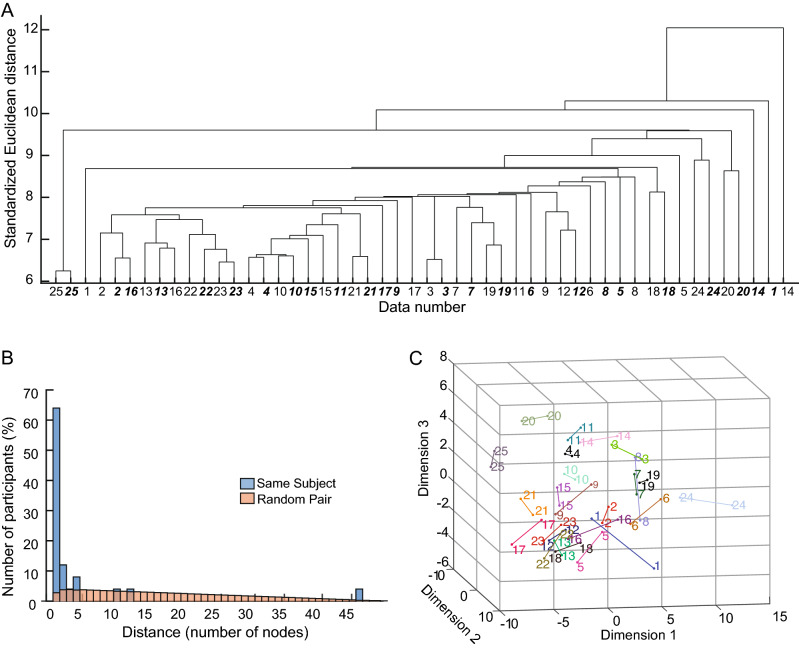
Cluster analysis results. (**A**) a dendrogram based on hierarchical cluster analysis with the standardized Euclidean distance and single-linkage method. Numbers on the edges indicate participants (#1-#25), and regular type is test 1 and bold, italic type is test 2 for each participant. (**B**) historgram of distance between test 1 and test 2 for each participant (blue) and random data pairs (light pink). Plots generated in Matlab version R2020a, https://matlab.mathworks.com. (**C**) Classical multidimensional scaling. Each number represents a participant (#1-#25) and lines join the two tests for each participant. Plot generated in Matlab version R2017b, https://matlab.mathworks.com.

Finally, we examined whether overall performance of individual subjects across tasks remained similar between repeat tests by using cluster analysis techniques. Figure 6A shows a dendrogram representing the result of single linkage clustering. Data of test 1 (normal font) and test 2 (bold, italic font) for the same participant are located next to each other (nearest neighbours) in 16 of 25 participants, indicating that overall performance was quite similar. A further 3 participants were located within 2 nodes (next nearest neighbours). Note that 11 of 13 last fusions with only two datasets paired were repeat tests from the same individual. The distribution of the distance between each data pair was much narrower compared to the distribution calculated by pairing random test data performed by different participants (Fig. 6B). Multidimensional scaling was used to visualize the location of each participant test pair in three dimensional space (Fig. 6C). There are few distinct clusters of participants with most of the participants in a central group. The notable exceptions are participants 25, 20 and 24 who are located outside the main cluster. These three participants are nearest neighbours on the cluster analysis. Most participant pairs that are nearest neighbours in the cluster analyses are also closely linked with multidimensional scaling.

## Discussion

The objective of the current study was to examine patterns of performance across five different motor tasks in a cohort of young adults. Correlations between spatial and temporal parameters within the same task were generally much higher than correlations across motor tasks. Further, cluster analysis found that repeat tests for the same individual tended to be grouped together.

Test–retest reliability in all tasks was assessed using ICC, where values above 0.75, 0.60 and 0.40 reflect ‘excellent’, ‘good’ and ‘fair’ repeatability, respectively^33^. In our cohort of young healthy adults, we found that most of the parameters for OH, RRM and TT were near or above 0.75. In some cases, lower ICCs were observed for VGR and BoB. For example, speed maxima count (r = 0.191) and initial speed ratio (r = 0.068) of VGR, or absolute reaction time difference (r = 0.130) and hand path length bias of BOB (r = 0.225). Low ICCs for this cohort of young adults indicates that there was not a large variation in performance across young, healthy participants as compared to the variation between repeat testing^34,35^. Many of these tasks were originally designed to quantify motor impairments in patient groups. Of note, since patient groups generally display a much greater range in performance^27,36^, ICC values tend to be much higher for patient cohorts. Thus, it is important to always assess the reliability of motor tasks in the participant cohort of interest.

Our observation of a higher number and a higher strength of correlations across spatial and temporal parameters within a given motor task is generally consistent with studies exploring data reduction techniques on the kinematics of motor performance^37–40^. A recent study^28^ demonstrated that principal component analysis (PCA) for a number of sensorimotor tasks could reduce the dimensionality of subject performance to 3 Principle Components (PC) and retain most of the variance. Further, the parameters associated with a given PC tended to reflect a specific aspect of motor performance such as the initial direction errors when reaching or features related to corrective movements during reaching. PCA has also been used to identify the patterns of high-dimensional motor parameters (e.g. kinematics). For example, effector-independent organization that governs the coordination of movements for reach and grasp^41^ or condition-dependent structures of hand postural variance during grasping and signing in sign language^42^.

We also found that correlations were relatively high between the left and right arms for a given task. VGR, RRM and TT all demonstrated similar patterns of high correlations across parameters across the two arms. This seems surprising given the substantive differences between arms in the performance of many motor skills, such as writing or throwing a ball; performance is clearly much better in the dominant arm, which is the right arm for ~ 90% of individuals^43–47^. In contrast, minimal differences in skills are observed in tasks such as typing. In some cases, better performance can be observed with the non-dominant arm such as skilled fingering on a stringed instrument. These examples highlight that skill is predominantly influenced by experience and training. The fact that we observed high correlations across arms for reaching (VGR), tracking (TT) and rapid reciprocal movements (RRM) likely reflect that these gross motor skills are performed with both limbs in daily activities. It also suggests that performance reflects a similar general strategy for performing each task regardless of the arm used to perform the task.

In contrast, we found fewer and weaker correlations across tasks, suggesting relative independence in how individuals perform different motor skills. The only parameter that tended to correlate across tasks was maximal speed which displayed significant correlations across VGR, BOB and RRM. Otherwise, surprisingly few parameter pairs reached significance across the five tasks. Further, our recent study found that PCA analysis across sensory and motor tasks demonstrated interactions within types of tasks, but limited interactions between different types of tasks^48^. Specifically, Principle Components (PCs) for reaching, rapid hitting and proprioceptive skills could include performance measures for the two arms, suggesting common strategies across the arms, but PCs were not found to span across these three types of motor skills. These results contrast with some studies investigating children’s motor skills which suggest that different motor skills are associated with each other, leading to a concept of general motor ability^11–13^. However, factors related to development, whether related to the musculoskeletal system or brain function, may have a substantive influence on motor skills of children. A study investigating the surgical skills of dentists showed that visual-spatial ability was associated with manual dexterity in novice trainees but not in experienced surgeons, suggesting that practice and experience may override or offset the influence of visual-spatial ability over time^49^.

Critically, our cluster analysis identified repeat tests for individual subjects tended to be nearest pairs across all 50 datasets. Over 60% (16/25) of individual subjects were located next to each other, with a further 3 individuals located within 2 nodes of each other (next nearest neighbours). Such a pattern is observable because individuals display a consistent strategy when performing the same task and display different strategies across tasks as compared to others. That is, two individuals that hit a similar number of objects (Total Hits) in OH, can have different Target Errors in TT. In effect, assessing subject performance across a range of behavioural tasks creates an individualized behavioural pattern.

This behavioural pattern across motor skills may be related to the idea of motor “style” or “signature”^4,6^. These ideas suggest that there are distinctive patterns or characteristics of movement by which an individual can be identified. This is commonly seen in movements such as walking^50^, cycling movements^51^ and arm movements^52^ and involve the identification of specific participants based on their kinetic or kinematic information, either visually or through automated search algorithms. For example, one study of over 100 walking participants was able to identify participants with > 99% accuracy based on foot plantar pressure profiles^10^. The same techniques were also able to successfully identify a subset of participants collected 5 years prior, suggesting a time invariant quality of the distinct motor patterns identified.

Perhaps the most intuitive example of motor signature or style is the recognized uniqueness of handwriting. Despite the fact that there are many ways to write each letter of the alphabet and to join the letters together to create words, handwriting is unique to each individual. Further, handwriting remains relatively invariant regardless of the size of the written letters and whether they are written by the dominant or non-dominant arm, using the foot or even the mouth, reflecting a common underlying strategy^53^. This unique invariant quality of handwriting is why it is used pervasively for legal, financial and other documents. Thus, if specific features of handwriting were quantified (i.e. the orientation, relative size and angle of the line when crossing the letter ‘t’), one would find high intraclass correlations from repeated writing of a word, as we did for specific parameters within each of our robot-based motor tasks.

Therefore, high intra-task correlations combined with low inter-task correlations that are repeatable across testing time points may be consistent with the idea of an ‘across-task’ behavioural signature for individuals. In other words, individuals have different levels of skill across tasks, forming a type of motor skill signature, which is consistent across testing points. Whereas previous literature has focused on classification of motor signatures within a specific task, we have extended this idea to include patterns of behavior across a set of five motor tasks. We hypothesize that such a behavioural signature should be even more robust with the addition of more behavioural tasks that quantify a broader range of sensory, motor and cognitive functions. While the ability to identify a nearest neighbor will decrease as individuals are added to the analysis, the expectation is that the relative distance will be much less than random chance so that an individual will remain within a small subspace across all task parameters. This behavioural pattern provides a potentially powerful approach for clinical assessment of brain function as substantive changes in the pattern of performance, even though still within the healthy range, may reflect a subtle but measureable change in decisional processing and motor control, indicative of early changes in brain function due to neurological disease or injury.

There are some limitations and future directions to consider with the present results. The present study was limited to 25 individuals, less than initially planned due to the outbreak of covid-19. However, the general pattern of high intra-task correlations and low inter-task correlations will not qualitatively change with a larger cohort, although specific correlations will become more precise as expected due to sample size. As well, repeat tests were performed in the same experimental session. The high intra-task correlations observed in the present study may be impacted by various factors such as the number of days between repeat assessments, fatigue and even time of day. However, as mentioned previously, plantar pressure profiles in participants performing a walking test remained similar up to 5 years apart^10^, suggesting that certain motor signatures may remain stable for years. Future work will explore how these factors influence intra- and inter-task correlations and thus the stability of behavioural patterns for individuals.

In conclusion, we found that in a healthy young adult cohort, motor skills used in different tasks are relatively independent, as we found low correlation between task parameters from different motor tasks. This finding, coupled with the consistency of the patterns of motor task parameters across repeated measurements found by the cluster analysis indicates that most individuals have different abilities across motor skills that are consistent across time points. Future studies will investigate factors (e.g. genetics, development, time between tests) that may contribute to shape these characteristics.

## Supplementary Information

Supplementary Figures.

## Data Availability

The datasets generated during and/or analysed during the current study are available from the corresponding author on reasonable request.
