# Supplementary material for: High intra-task and low inter-task correlations of motor skills in humans creates an individualized behavioural pattern
Source: Sci Rep. 2022 Nov 23;12:20156. doi: 10.1038/s41598-022-24479-w (PMC9684559; doi:10.1038/s41598-022-24479-w)
Supplement: Supplementary file 1 — Supplementary Figures. [file 41598_2022_24479_MOESM1_ESM.pdf]

Supplemental Material

High intra-task and low inter-task correlations of motor skills in humans creates an individualized behavioural pattern

Shoko Kasuga[1], Ethan Heming[1], Catherine R. Lowrey[1], Stephen H. Scott*[1,2,3,4]

[1]*Centre for Neuroscience Studies, Queen's University, Kingston, Ontario K7L 3N6, Canada*

[2]*Department of Biomedical and Molecular Sciences, and*

[3]*Department of Medicine, Queen's University, Kingston, Ontario K7L 3N6, Canada*

[4]*Kinarm, BKIN Technologies Ltd. Kingston, ON, Canada.*

Corresponding author

Stephen H. Scott, PhD

Room 219, Botterell Hall, Queen's University, Kingston, Ontario K7 L 3N6, Canada.

E-mail: steve.scott@queensu.ca

Task Parameter Calculation for Rapid Reciprocal Movement task and Target Tracing task

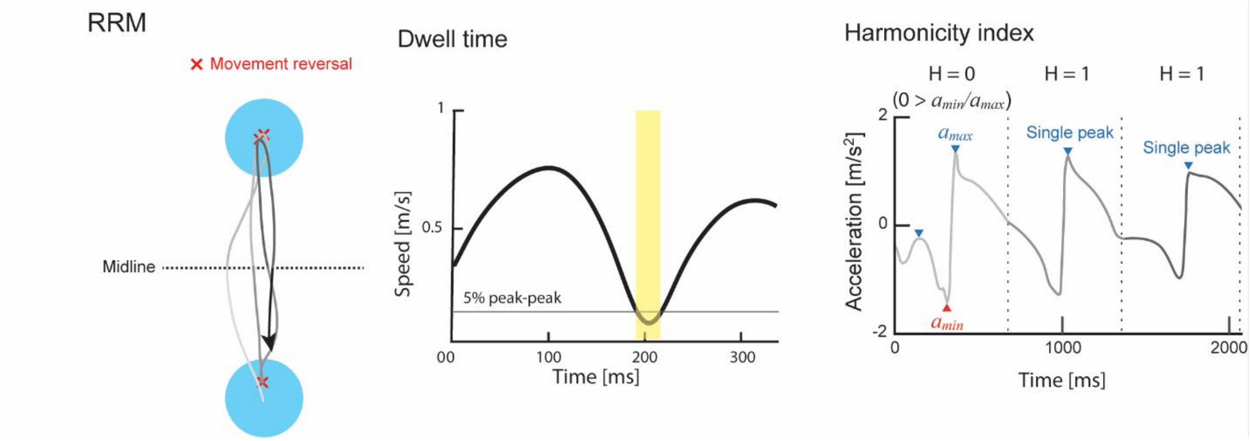

Supplementary Figure 1: Task parameters for RRM. Left panel, hand trajectories of successive cycles. Movement reversals were defined as the points where participants made turns of movements (red cross marks). A full cycle was defined as the time when the hand passed the midline (dashed line) until the hand passed the midline in the same direction (i.e., reach out-and-back-and-out). Middle panel, dwell time. Duration around movement reversal between the first time the speed decreases below 5% of the difference between speed minima and maxima of the preceding movement, and the first time it increases above 5% of the that for the following movement (yellow shaded area). Right panel, Harmonicity index. Index computed by the hand acceleration (a) reflecting the symmetry of hand motion around movement reversal, i.e., when the hand passed midline between the target (before movement reversal) and the hand returned to the midline (after movement reversal). If there is a single acceleration peak in this time window, i.e., an ideal rhythmic movement with sinusoidal speed profile, H = 1. If there are multiple acceleration peaks, H is calculated by the following equation:

$$H = \max(\frac{a_{min}}{a_{max}}, 0)$$

Plots generated in Matlab version R2020a, https://matlab.mathworks.com

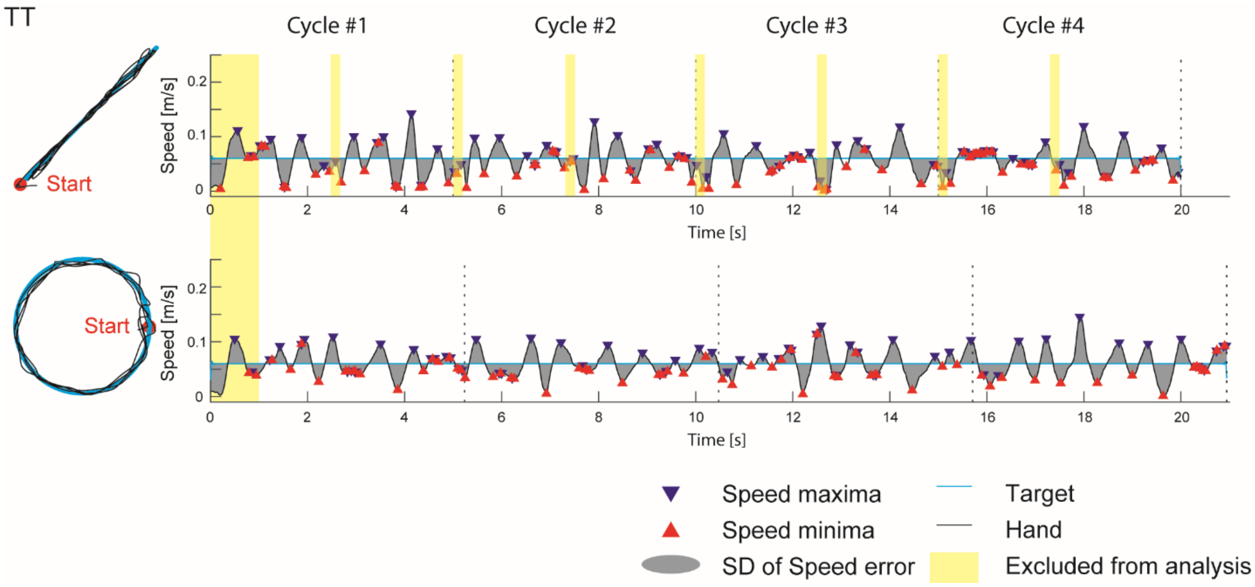

Supplementary Figure 2: Task Parameters for the Tracing Task. Upper row, hand trajectories in the target motion along the diagonal direction (left) and hand speed across four cycles (right). Lower row, hand trajectories in the target motion along the circle (left) and hand speed across four cycles (right). In the left panels, path length ratio is defined as the ratio of the distance travelled by the hand (black traces) in a trial and the distance travelled by the moving target (cyan traces). Target error was defined as mean distance between hand and moving target. In the right panels, target speed is indicated by blue lines, hand speed is indicated by black lines, speed maxima are indicated by blue triangles, and speed minima are indicated by red triangles. Min-max speed difference is mean difference between pairs of adjacent speed minima and maxima, for all such pairs in a trial. SD of speed error is difference between hand and target speed (gray shaded areas). The initial 1000ms after target motion was initiated (both circle and line objects) and 200ms after reversals (line objects only) was excluded from the analyses (yellow shaded area). Plots generated in Matlab version R2020a, https://matlab.mathworks.com

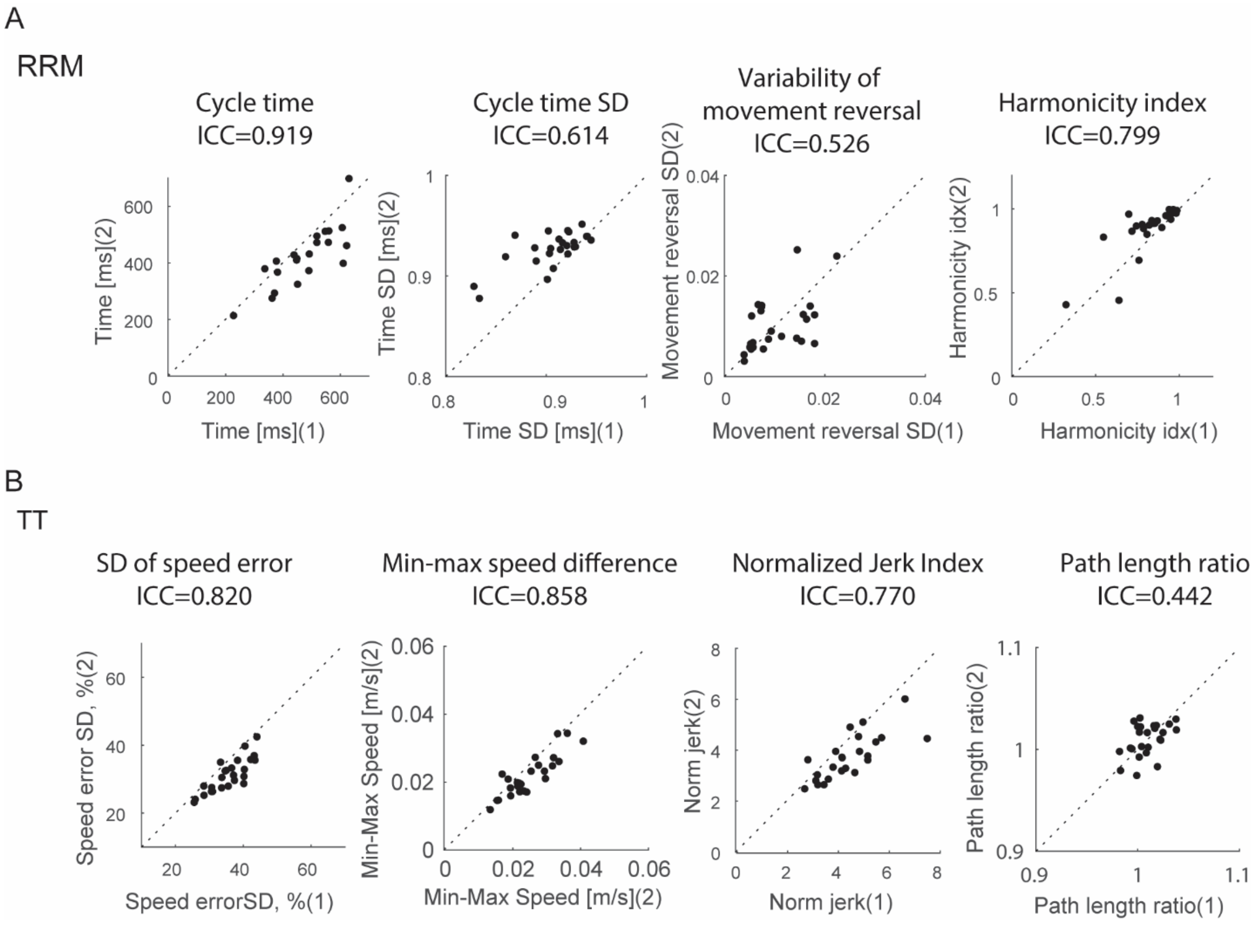

Supplementary Figure 3: Test-retest reliability for RRM and TT. A, scatter plots of the relationship between four example parameters in Rapid Repeated Motion (cycle time, cycle time SD, variability of movement reversal, harmonicity index) with ICC values. Each data point represents the task parameter calculated for a single subject at test 1 and test 2. B, scatter plots of the relationship between four example parameters in Target Tracking (SD of speed error, min-max speed difference, normalized jerk index, path length ratio) with ICC values. Each data point represents the task parameter calculated for a single subject at test 1 and test 2. Plots generated in Matlab version R2020a, https://matlab.mathworks.com
